# Exploring Tumor–Immune Interactions in Co-Culture Models of T Cells and Tumor Organoids Derived from Patients

**DOI:** 10.3390/ijms241914609

**Published:** 2023-09-27

**Authors:** So-Ra Jeong, Minyong Kang

**Affiliations:** 1 Department of Urology, Samsung Medical Center, Sungkyunkwan University School of Medicine, Seoul 06531, Republic of Korea; wjdxmfhf@naver.com; 2 Department of Health Sciences and Technology, The Samsung Advanced Institute for Health Sciences & Technology (SAIHST), Sungkyunkwan University, Seoul 06355, Republic of Korea; 3 Samsung Genome Institute, Samsung Medical Center, Seoul 06531, Republic of Korea

**Keywords:** patient-derived organoid, T cells, co-culture system, tumor microenvironment

## Abstract

The use of patient-derived tumor tissues and cells has led to significant advances in personalized cancer therapy and precision medicine. The advent of genomic sequencing technologies has enabled the comprehensive analysis of tumor characteristics. The three-dimensional tumor organoids derived from self-organizing cancer stem cells are valuable ex vivo models that faithfully replicate the structure, unique features, and genetic characteristics of tumors. These tumor organoids have emerged as innovative tools that are extensively employed in drug testing, genome editing, and transplantation to guide personalized therapy in clinical settings. However, a major limitation of this emerging technology is the absence of a tumor microenvironment that includes immune and stromal cells. The therapeutic efficacy of immune checkpoint inhibitors has underscored the importance of immune cells, particularly cytotoxic T cells that infiltrate the vicinity of tumors, in patient prognosis. To address this limitation, co-culture techniques combining tumor organoids and T cells have been developed, offering diverse avenues for studying individualized drug responsiveness. By integrating cellular components of the tumor microenvironment, including T cells, into tumor organoid cultures, immuno-oncology has embraced this technology, which is rapidly advancing. Recent progress in co-culture models of tumor organoids has allowed for a better understanding of the advantages and limitations of this novel model, thereby exploring its full potential. This review focuses on the current applications of organoid-T cell co-culture models in cancer research and highlights the remaining challenges that need to be addressed for its broader implementation in anti-cancer therapy.

## 1. Introduction

Recent progress in our understanding of the tumor microenvironment (TME) has led to the development of effective therapies for advanced cancer in recent years. Treating numerous patients with cancer with monoclonal antibodies that target the inhibitory receptors expressed by immune cells (immune checkpoint blockade, ICB) has shown remarkable response rates in various solid tumors and hematologic malignancies [1]. Therefore, it is crucial to develop preclinical models that can investigate the TME and guide clinical precision therapy [2]. Immune checkpoint inhibition (ICI) therapy has emerged as a groundbreaking advancement in cancer treatment, revolutionizing the field. Over the past decade, anti-programmed cell death protein 1 (PD-1) and anti-cytotoxic T-lymphocyte-associated protein 4 (CTLA-4) drugs, such as nivolumab and ipilimumab, have demonstrated remarkable clinical efficacy in specific patient groups [3,4]. Numerous clinical trials have provided substantial evidence supporting the effectiveness of these therapies [5]. They have shown particularly high efficacy in tumor types characterized by high mutational burden resulting from genetic instability [6]. Although therapies focusing on ICI show promising clinical potential, their effectiveness, particularly in solid tumors, remains limited [7]. Despite improvements in patient outcomes with ICI therapies for various cancer types, only a small percentage of patients treated with ICI achieve long-lasting responses. Even in melanoma, which has a relatively high response rate to ICI, 60–70% of patients do not experience a significant response to anti-PD-1 therapy. Among those who respond, 20–30% eventually experience tumor relapse and progression [8,9,10]. These findings highlight the need for further research to understand the underlying mechanisms of resistance to ICI therapy and to develop strategies to improve treatment responses and long-term patient outcomes. Tumor organoids are three-dimensional (3D) in vitro cultures derived from patient tumor samples that accurately represent the genetic features and histological properties of the original tumor [11,12]. These organoids contain various cell types, including differentiated and cancer stem cells, faithfully mimicking the composition of native tissue [13,14]. However, the lack of representation of the TME, including the T cells surrounding the tumor in in vitro models, leads to discrepancies between the clinical response to ICI administered to patients and the observed responsiveness in preclinical settings [15,16]. Although precision 3D in vitro models have been successfully implemented for certain cancer types (e.g., breast cancer [17] and liver cancer [18]), their use in the preclinical modeling of pancreatic cancer has been largely limited to patient-derived mouse xenografts. Patient-derived mouse xenograft models have primarily been used for the preclinical evaluation of therapeutic agents, and the success of engraftment is correlated with the extent of prior treatment [19]. Tumors that have undergone extensive pre-treatment often fail to successfully engraft as xenografts. Therefore, recent attempts have been made to co-culture tumor organoids and T cells to mimic the interactions between tumor and T cells [20]. Co-culture models of T cells and tumor organoids provide valuable tools for investigating these interactions and understanding the mechanisms of tumor immunity. This review aims to critically evaluate the current state of knowledge on co-culture models of T cells and tumor organoids, identify gaps in the literature, and provide recommendations for future research.

## 2. Overview of Co-Culture Models in Cancer Research

**Figure 1:**
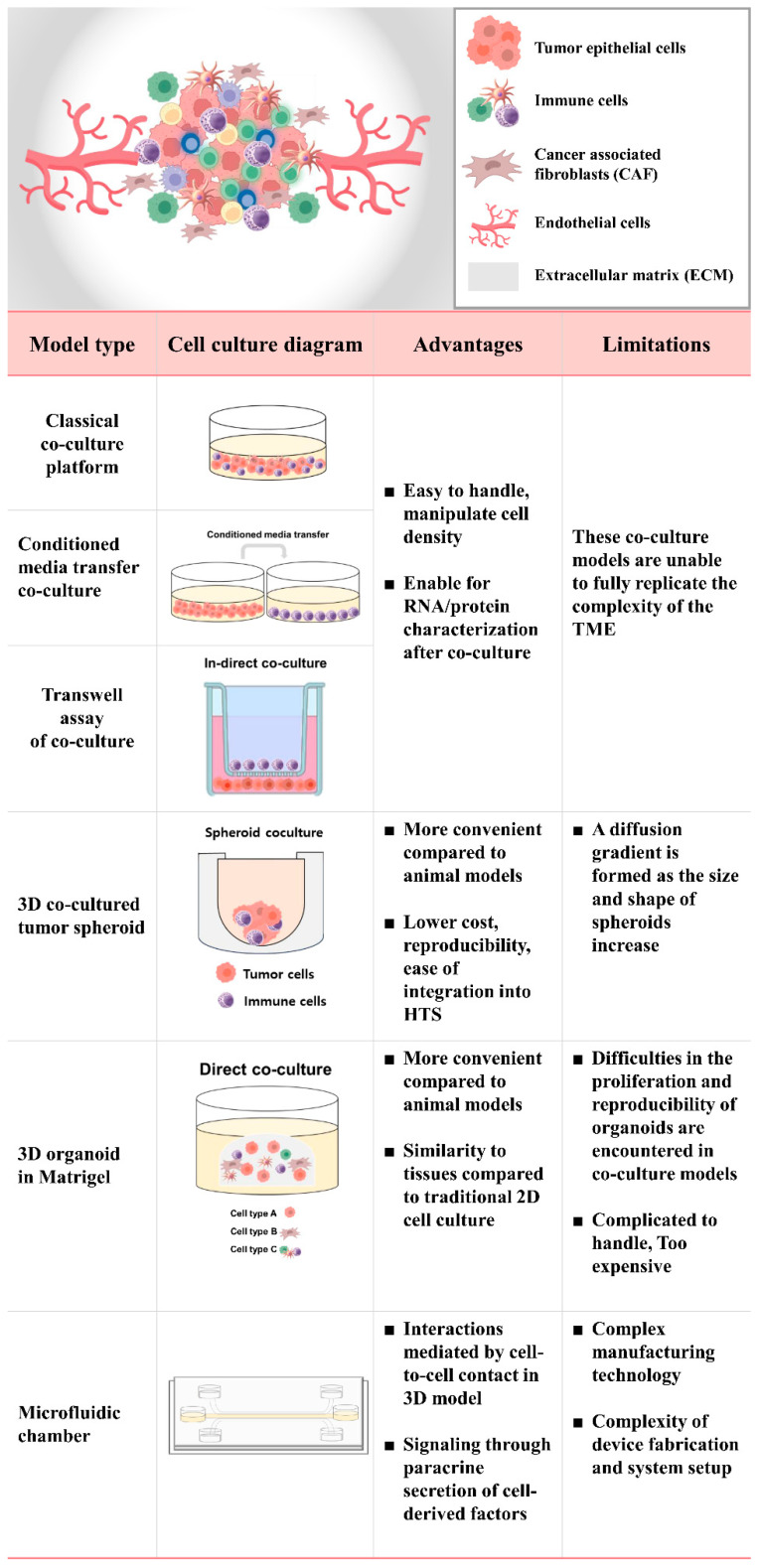
Various types of 2D and 3D in Vitro Cell Culture Models. The classical co-culture platform involves embedding 2D cancer cell lines and immune cells into a cell culture dish. Media can be transferred to another dish for this purpose. In the transwell co-culture system, immune cells are embedded in a matrix on top of a transwell insert, while cancer cells are embedded in the bottom. A 3D spheroid co-cultured with immune cells represents a 3D cell culture model where cells aggregate to form a spherical structure. The integration of organoids, immune cells, and cancer-associated fibroblasts (CAFs) is achieved by embedding them in Matrigel. Microfluidic chambers, also referred to as organ chips, are miniature devices designed to mimic the functions of human organs on a microscale. These chips are typically constructed from transparent materials like silicone or glass and feature tiny channels or chambers that can be lined with human cells. The primary component of these microfluidic chambers is the irrigation-controlled microchannel, which supports the growth of various living cells such as cell lines, immune cells, and organoid endothelial cells.

Co-culture models in cancer research have personalized medical applications [21,22]. Co-culture systems can be used to investigate the role of immune cells in tumor progression, the impact of immune cell infiltration on tumor growth and metastasis, the effectiveness of immunotherapies, and the development of drug resistance mechanisms [23,24,25]. Co-culture models have been developed for various types of cancer, including solid tumors and hematological malignancies [26,27]. Immunotherapy has significantly improved the overall survival of patients with cancer, particularly those with hematological malignancies [28]. The co-culture models provide a powerful experimental platform for studying the complex interactions between tumor cells and various immune cells, advancing our understanding of cancer biology, facilitating the development of immunotherapeutic strategies, and enabling high-throughput screening [29,30]. These models include various types of co-culture systems, such as direct or indirect co-cultures [31], media transfer models [32], transwell co-cultures [33], 3D organoid co-culture systems [34], and microfluidic chambers [35], each with its advantages and limitations (Figure 1). The current methods of preclinical discovery for new anti-cancer therapeutics involving two-dimensional (2D) in vitro cell cultures and animal models face challenges such as high costs, ethical concerns related to rodent use, and low correlations with clinical trial data owing to species differences. Moreover, 2D cell cultures do not accurately represent the 3D spatial environment, extracellular matrix, or stromal cellular components of human tumors. To address these limitations, researchers have turned to 3D in vitro tumor models as potential alternative testing platforms for screening new anti-cancer drugs [36].

Cell spheroids have gained significant attention among the different types of 3D tumor models and are widely used as scaffold-free models. Cell spheroids are aggregates of cancer cells with tumor-like characteristics [37]. They provide a more realistic representation of the TME than 2D cultures. Several technologies are available for the assembly of 3D spheroids. These models offer a more representative simulation of the complex TME and aid in evaluating drug responses and potential immunotherapy strategies [38]. Spheroids are structurally simple models that are primarily used for applications such as drug screening. Organoids, however, derived from stem or progenitor cells, are cellular aggregates used to miniaturize and replicate organ functions. They have gained increasing prominence in developmental biology and medicine. Organoids closely resemble the histological and genetic characteristics of the original tumor from which they are derived. Due to their ease of generation, long-term culture capabilities, and the ability to cryopreserve them, organoids have become increasingly important in fields such as cancer research, neurobiology, stem cell research, and drug development, as they offer an enhanced modeling of human tissues [33]. 

Organoid-on-a-chip denotes an experimental model employed in scientific and medical research, combining two technologies: organoids and microfluidic chips (microchips). These models are capable of replicating dynamic microenvironments, including those found in tumor pathophysiology and tissue-tissue interactions [39]. Organoids have typically been cultured in a substance called Matrigel. Matrigel is derived from the secretions of Engelbreth-Holm-Swarm mouse sarcoma cells and is complex and poorly defined [40]. This complexity makes it challenging to understand the specific factors of Matrigel that regulate organoid development. Interestingly, according to research by Tsai and colleagues, after 72 h of cultivation in the liquid phase of organoid cultures, T-lymphocytes remained viable. Interestingly, those T-lymphocytes situated at the periphery of empty Matrigel domes formed a distinct boundary without infiltrating the Matrigel substance. In contrast, when T cells were positioned at the boundary of Matrigel domes containing patient-derived primary organoids, they exhibited infiltration into the Matrigel, migrated towards the organoids, and diffused along the boundary. Notably, lymphocyte infiltration was observed exclusively in the presence of organoids. These findings strongly suggest that T cell migration in these innovative organotypic models is influenced by the presence of tumor cells. T cells play a crucial role as major effectors of the immune response against tumors. When activated by tumor-associated antigens, they can proliferate and exert cytotoxic effects, eliminating tumor cells. However, immune escape mechanisms in tumors, including the overexpression of immune checkpoints, such as PD-1, contribute to tumor-related immunosuppression. Current immunotherapies targeting these pathways have shown limited efficacy in colorectal cancer (CRC), highlighting the need to identify new targets for immunotherapy in this cancer type. Bonnereau J et al. [41] investigated the phenotype of tumor-related and non-tumor-related intestinal T cells in a prospective cohort of patients with CRC (n = 44), focusing particularly on the adenosinergic pathway and its correlation with the clinical phenotype. To create a relevant experimental model, they developed an autologous co-culture system using patient-derived primary tumor spheroids and their tumor-associated lymphocytes. Using this model, it is possible to assess the impact of CD39 inhibition on the immune response of anti-tumor T cells. These results revealed an increased expression of CD39, along with its co-expression with PD-1, in tumor-infiltrating T cells compared with that in mucosal lymphocytes. CD39 expression was higher in the right colon and early-stage tumors, suggesting that a subset of patients may benefit from a CD39 blockade. Moreover, under autologous conditions, they demonstrated that blocking CD39 induced T cell infiltration and led to the destruction of tumor spheroids in the co-culture system. In recent years, significant efforts have been devoted to developing 3D co-culture tumor models involving the co-cultivation of patient-derived organoids (PDO) and T cells [41]. Cellular immunotherapy has reshaped the landscape of therapeutic oncology. One notable approach is chimeric antigen receptor (CAR) T cell therapy, a technique involving the collection of an individual’s T cells and genetically engineering them to express CARs capable of recognizing and attacking cancer cells [42]. Typically, CAR T cells are administered systemically to target tumor cells and exert their anti-tumor activity [43]. Organoid-immune cell co-culture models have been validated as crucial preclinical models for assessing the efficacy and toxicity of immunotherapies for solid tumors. In cases where the development of CAR T cells has been hindered by off-target toxicities related to antigen expression in normal tissues, these models have provided crucial insights. Schnalzger et al. (2019) developed experiments to evaluate the killing capacity of CAR-engineered natural killer cells against both normal and tumor-derived PDOs. Their research demonstrates the suitability of organoid models for validating tumor-specific neoantigen targets and identifying off-target toxicities in a preclinical context [44]. More recently, Dekkers et al. (2023) have developed a co-culture system to observe the real-time activity and behavior of engineered T cells against PDOs [45]. Immune-compromised models fail to replicate the intact immune system, thus limiting their ability to regulate CAR T cell function. Furthermore, there is clinical evidence of crosstalk between infused CAR T cells and the endogenous immune system [46,47], emphasizing the need to analyze CAR T cells in an immune-competent environment.

Organoids self-assemble into intricate structures that partially mimic in vivo physiology, making them promising tools for bridging the gap between preclinical research and clinical applications. Consequently, organoids have emerged as a valuable resource for in vitro drug testing [48]. Organoids are anticipated to facilitate advancements in understanding diseases that have historically been challenging or impossible to model accurately.

## 3. Co-Culture Models of T Cells and Tumor Organoids

**Figure 2:**
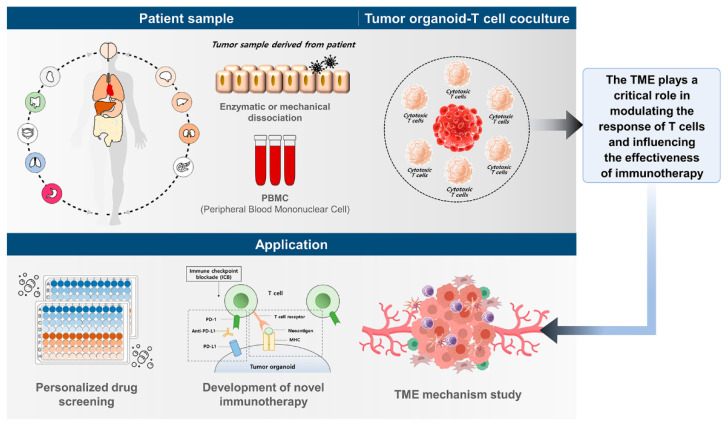
Schematic diagram of patient-derived 3D organoid–T cell co-culture models and application. Tumor tissues are collected after surgical resections to isolate TILs and generate organoids from patients. Additionally, autologous PBMCs can be isolated from the blood. The co-culture model of organoids and T cells is optimized for studying disease modeling, patient-specific drug responsiveness, and signaling pathways mediated by interactions in the TME, representing biological relevance. It can be applied in various research areas.

Cancer organoids serve as valuable ex vivo and in vitro model systems to study the impact of the tumor microenvironment (TME) on cancer growth. In recent years, co-culture models of T cells and tumor organoids have gained popularity owing to advancements in organoid technology and the increasing interest in cancer immunotherapy research [49,50]. Efforts have been made to co-culture tumor organoids with various immune cell types (Table 1). These models contribute to our understanding of tumor immunology, aid in developing novel immunotherapies, and facilitate personalized medical approaches by assessing individual responses to treatments (Figure 2). A published protocol from 2021 provides a useful tool for co-culturing human intestinal organoids and CD4^+^ T cells to investigate T cell–intestinal epithelial cell interactions during tissue development and inflammation [51]. One approach involves co-culturing cancer organoids with peripheral blood mononuclear cells (PBMCs) to generate patient-specific tumor-reactive cytotoxic T cells [20]. This strategy takes advantage of the fact that a high level of neoantigen presentation is crucial for eliciting a strong anti-tumor immune response mediated by antigen-specific T cells. Dijkstra et al. [52] successfully expanded tumor-reactive T cells from paired PBMCs using microsatellite instability (MSI)-high CRC and non-small cell lung cancer (NSCLC) organoids. They quantified the expansion of T cells based on organoid-specific killing. In co-cultures of CRC T cell organoids with high major histocompatibility complex-I expression, CD8^+^ T cells showed an upregulation of CD107a and a secretion of IFN-gamma in 50% of the cases. Similar responses were observed in NSCLC co-cultures, even in patients who did not exhibit tumor reactivity before the co-culture. One study used co-cultures to demonstrate PBMCs and purified T cell responses against patient-derived cholangiocarcinoma to study growth inhibition and the induction of organoid cell death by these cells [53]. Furthermore, they investigated the mechanism of cell death induced by T cells and described patient-specific differences in the sensitivity to immune cell cytotoxicity. Meng et al. [54] developed a platform that involved co-culturing autologous tumor organoids with PBMCs to identify and expand tumor-targeting T cells from the circulation of patients with pancreatic cancer. T cells pre-treated with these organoids underwent clonal expansion and expressed tissue-resident memory T cell markers, demonstrating their ability to effectively kill autologous tumor organoids. These findings suggest the potential of co-cultures to generate tumor-reactive T cells, indicating the possibility of personalized immunotherapy approaches. The extent of cell death observed in an in vitro co-culture assay correlated well with the patient’s response to chemotherapy and immune checkpoint blockades [55]. Additionally, the authors tested the effectiveness of ICB using PD-1 antibodies in a co-culture model and found that blocking PD-1 improved organoid killing by PD-1^high^ T cells. Two other groups have described organoid-based immuno-oncology assays that involve engineered cytotoxic lymphocytes designed to recognize specific antigens and kill organoids expressing these antigens [44,55]. The first group engineered chimeric antigen receptor-modified natural killer cells to recognize selected antigens [44], whereas the second group generated cytotoxic T cells with transgenic T cell receptors (TCRs). In both cases, a robust antigen-specific cytotoxicity was observed against cancer organoids that presented the target antigen [44]. Co-culture models of T cells and tumor organoids offer a versatile platform for investigating the intricate interactions between T and tumor cells. By incorporating various factors, such as the presence of other immune cell populations, the role of tumor-associated antigens, and the effects of immune-modulating drugs, these models enable the exploration of their impact on T cell–tumor interactions. Moreover, these models serve as valuable tools for evaluating the effectiveness of novel therapies or combination treatments in a clinically relevant context, aiding the advancement of cancer research and the development of more efficient cancer treatments.

## 4. Clinical Relevance of Co-Culture Models of T Cells and Tumor Organoids

This immune–organoid co-culture platform can potentially advance immunotherapy research based on individualized patient characteristics. One treatment approach that can benefit from personalized strategies is ICB therapy, which relies on the interaction between immune checkpoints and their corresponding ligands on immune and tumor cells. Because of the intra- and inter-tumoral heterogeneities in the clinical efficacy of ICB therapy, Votanopoulos et al. [77] investigated the effects of the PD-1 inhibitors pembrolizumab and nivolumab on organoids. They observed a decrease in cell viability in organoids made from a composite of patient-derived melanoma cells and lymph nodes. In 85% of the cases (six out of seven), immune-enhanced patient-derived tumor organoids (iPTOs) showed a good response to immunotherapy, consistent with the clinical response observed. In another experimental study, T cells from PBMCs were circulated through iPTO and subsequently transferred to tumor organoids from the same patient, resulting in tumor killing, suggesting a possible role of iPTO in generating adaptive immunity. In the study by Chalabi et al. [78]., a co-culture model was constructed using PBMCs from a cohort of patients with early-stage colon cancer who received combined anti-PD-1 and anti-cytotoxic T-lymphocyte-associated protein 4 neoadjuvant immunotherapy. Six non-responders and six responders were selected for this study. The in vitro experiments demonstrated that T cells from responders were activated and effectively killed tumor cells, whereas T cells from non-responders showed no reactivity toward tumor organoids. Three of the six responder patients did not exhibit T cell reactivity. This suggests that the co-culture model requires further optimization to improve its accuracy in predicting the efficacy of ICI therapy. Teijeira et al. [79] established seven PDOs from treatment-resistant metastatic CRC and one PDO from treatment-naïve primary CRC. This study examined the sensitivity mechanisms of cibisatamab, a bispecific monoclonal antibody that targets intratumoral carcinoembryonic antigen (CEA), in relation to CD3 T cell activation Researchers have used a co-culture system with PDOs and allogeneic CD8^+^ T cells to evaluate the efficacy of cibisatamab. The results showed that PDOs with a low CEA expression exhibited resistance to cibisatamab, whereas those with a high CEA expression were sensitive to the antibody. Using organoids in co-culture models provides valuable insights into the resistance of MSI-high CRC to ICI in the presence of inflammation. Sui et al. established tumor organoids derived from MSI-high CRC patients who received PD-1 blockade therapy and co-cultured them with TILs or PBMC-derived T cells. The results showed that patients with local inflammatory conditions during treatment exhibited a higher proportion of disease progression and worse progression-free survival [80]. Furthermore, the co-culture of organoids with immune cells demonstrated a higher proportion of apoptotic organoid cells in the PBMC group in one patient with inflammation, suggesting an inhibited local immune response to tumors [81]. Previous studies have shown that inhibiting PI3K can enhance the effectiveness of immunotherapy by sensitizing tumor cells to immune recognition or improving T cell functions [82]. Additionally, KRAS mutations are associated with promoting inflammation and the secretion of immunosuppressive cytokines [83]. In this study, the researchers investigated whether the combination of PI3K and immune checkpoint inhibitors influenced T cell-mediated tumor killing in a co-culture system using patient-derived tumor organoids (PDTOs). They found that the pre-treatment of PDTOs with PI3K inhibitors resulted in a reduction in IL-8 secretion, a cytokine that promotes NSCLC cell growth and survival, and hinders T cell function by upregulating PDL-1 on tumor cells and inducing apoptosis in certain CD8^+^ T cell subsets [64]. As preclinical models, PDTOs are still in the early stages of development, particularly for therapies that require the presence of immune cells. It is crucial to validate PDTO responses against patient responses to confirm the usefulness of these co-culture platforms as preclinical models for immunotherapy. Although there is a long road ahead and room for improvement, these models will undoubtedly positively affect precision cancer immunology in the coming years.

## 5. Challenges and Limitations of Co-Culture Models of T Cells and Tumor Organoids

Although organoids are recognized as “miniature organs” and show great potential in basic cancer research and clinical applications, several challenges and obstacles still need to be addressed. First, the establishment, maintenance, and transplantation of organoids can be costly [84]. Second, the success rates of establishing different types of cancer organoids vary considerably. Improving the establishment rate is important and can be influenced by various factors, such as the cellular composition of the original tissues [85]. Third, it is necessary to establish optimized and standardized culture conditions for different tumor organoids to enhance their reproducibility on a large scale and facilitate their application in HTS [86]. In addition, intra-tumoral heterogeneity contributes to drug resistance, compromising cancer treatment efficacy. Using established colon organoid and cell line models, drug responses were measured for 24 anti-cancer compounds. All 12 patients exhibited diverse molecular heterogeneity based on tumor regions, and even within the same tumor region, significant differences in drug responses were observed among different subregions [87]. In particular, organoids established from different regions within a single tumor show varying responsiveness to the same molecularly targeted anti-cancer agents, depending on their mechanisms of action. To overcome this challenge, it is important to target mutations shared by all cancer subclones and comprehensively integrate various genetic factors, transcriptomes, and protein heterogeneity. Obtaining patient-derived tumor tissues and immune cells such as TILs can be challenging. Heterogeneity among patient samples and the limited availability of these materials can affect the reproducibility and scalability of co-culture models. Co-culture models often utilize isolated immune cell populations such as T cells or PBMCs, which may not fully represent the complexity of the entire immune system. The interactions and contributions of other immune cell subsets, such as dendritic cells, macrophages, and natural killer cells, are not fully understood in these simplified models [25]. 

Although it partially reflects interactions between tumor and immune cells, it cannot fully replicate the intricate mechanisms and interactions found in the TME, including those among surrounding cells such as vascular cells and CAFs. Consequently, co-culture models require further refinement to better address the complexity and diversity of tumor-immune interactions, as well as to tackle issues related to standardization and reproducibility. 

## 6. Future Directions for Co-Culture Models of T Cells and Tumor Organoids

To enhance the efficacy of anti-tumor immunotherapy, there is a growing focus on testing the immune response of patients using in vitro microfluidic technology, also known as organ-on-a-chip technology. These systems, based on human microfluidic chips, aim to assess the effects of ICB on a controlled and representative TME [23]. A lymph node-on-a-chip flow device was developed to investigate the mechanical forces between antigen-presenting dendritic cells (DCs) and different types of T cells (CD4^+^ versus CD8^+^) or antigen-specific and non-specific T cells in in vitro settings. This device utilizes a simple perfusion system that applies a controlled tangential shear, allowing the study of interactions that are not easily detectable using traditional in vitro systems [87]. Aung et al. [88] employed a tumor-on-a-chip platform based on GelMA gelatin hydrogel to develop a breast cancer model. This ex vivo platform incorporates multiple cell types, including cancer cells (MCF7), monocytes (THP-1), and endothelial cells. To simulate this process, T cells were dispersed in perfused media and allowed to infiltrate the tumor model. The addition of monocytes to cancer cells improves T cell recruitment, which is associated with chemokine secretion. This microfluidics-based approach enables the modeling of 3D tumor tissues with microenvironmental heterogeneity, thereby providing valuable cancer models for various clinical applications. Further studies are needed to validate the clinical applicability of these novel tumor models, considering patient response data, and to evaluate their suitability for immunotherapy screening purposes.

Organoids have emerged as a groundbreaking technology and become vital methodologies in biomedical studies. They have found applications in tissue engineering, regenerative medicine, disease modeling, drug screening, and toxicological studies, allowing for the restoration of 3D structures and primary cell types. Furthermore, they have been used in translational applications, such as predicting chemotherapy and radiotherapy resistance before treatment, and gene editing for mutation rectification [89]. Although organoids have diverse applications in cancer research and clinical practice, their current version represents a preliminary model, and there is a constant need for the standardization and improvement of culture procedures that are specific to different cancer types. Organoids have been successfully generated from various organs, including the brain, retina, gastrointestinal tract, tongue, thyroid, liver, pancreas, skin, lungs, kidneys, and heart [64,90,91,92,93,94,95,96,97,98]. Three-dimensional organoid models have been established to investigate most cancers without significant technological limitations. Tumor cell lines in mice and patient-derived xenografts have been widely used as cancer research models, yielding valuable insights. However, these models have limitations that hinder their clinical applications. Cell lines typically lack the complexity of co-cultured immune cells, stromal cells, the TME, and organ-specific characteristics, leading to a loss of genetic heterogeneity over time and clonal selection. Xenograft models are time- and resource-intensive. Cancer organoids offer a promising solution for overcoming these limitations. Genetically modified organoids can be generated by genetically modifying stem cells to harbor oncogenic mutations. Unlike patient-derived xenografts, cancer organoids are more accessible and can be stored in biobanks for future use, enabling high-throughput drug screening. Cancer organoids that are specific to certain types of cancer or individual patients hold great potential as powerful tools for precision therapy. Additionally, biobanks of cancer organoids can be utilized for drug discovery and exploring new indications. However, cancer organoids are limited by the scarcity of immune cells and the specific types of stromal cells associated with cancer. Nonetheless, this approach enables researchers to capture the complexity of the TME. Co-culturing cancer organoids with immune cells offers a valuable model system for assessing the sensitivity of individual cancers to immunotherapy at any stage of treatment. It also provides a clinically applicable approach for generating patient-specific T cell products for adoptive T cell transfer. By co-culturing circulating tumor-reactive T cells with cancer organoids, it is possible to expand and enhance the functionality of these T cells. NK cells, T cells, and dendritic cells were co-cultured with cancer organoids to investigate immune responses in the context of cancer. This innovative model has proven effective for rapidly evaluating the impact of ICI on activating cytotoxic lymphocytes and promoting T cell infiltration, particularly within the context of T cell infiltrates. In addition, single-cell T cell receptor sequencing (scTCR-seq) offers a novel approach for the identification of paired α- and β-TCR subunits that determine the specificity of infiltrating T cells. Combining scTCR-seq with the analysis of T cell phenotypes (activation, memory, and exhaustion) and antigen specificity can provide more comprehensive insights into cancer immunotherapy [99]. This emerging tool has great potential for advancing our understanding of cancer immunotherapy and improving its effectiveness.

## 7. Conclusions

Personalized medicine is becoming increasingly important because of a deeper understanding of the diversity of tumor-infiltrating cells and intra-tumoral heterogeneity. This complexity is recognized because immunotherapeutic strategies are often perceived as effective only in a subset of patients. Co-culture models of tumor and immune cells enhance our understanding of tumor–immune interactions and, more importantly, serve as tools to assess patient-specific responses before immune therapy. Furthermore, these models have additional applications, such as transferring expanded tumor-reactive lymphocytes that are specific to individual patients and discovering neoantigens for vaccine development. Overcoming the limitations of co-culture models is crucial to gain a deeper understanding of patient-specific drug responsiveness. Our goal is to provide foundational information for developing valuable co-culture models of tumor organoids and T cells and to demonstrate their potential utility in exploring critical questions related to cancer biology. By proposing these models, we aim to address the limitations of current approaches and highlight the importance of investigating the TME for effective precision cancer therapy. We believe that, as co-culture models become more widely available, comprehensive genomic analyses of the TME will quickly follow suit, leading to valuable discoveries and advancements in cancer research.

## Figures and Tables

**Table 1:** Comparison of organoid microenvironment models and co-culture techniques.

Tissue Origin	Organoid Success Rate	Co-Culture-Immune Cell	Co-Culture Method	Drug Test	References
Colorectal cancer	80%	Dendritic cells CD8^+^ T cell, macrophage	Domes Matrigel	Immunotherapy	[20,56,57]
Renal cell carcinoma	67%	CD8^+^ T cell	Submerged Matrigel, ALI system, a 3D spheroid co-culture system	Immunotherapy	[58,59]
Lung cancer	79%	CD4^+^ T cell, CD8^+^ T cell, macrophages	Submerged Matrigel domes, ALI system	Immunotherapy, CART cell therapy	[21,60,61,62]
Bladder cancer	50%	CD8^+^ T cell	Domes Matrigel	Immunotherapy	[63,64,65,66]
Pancreatic cancer	75–83%	CD8^+^ T cell, Dendritic cell	Domes Matrigel	Immunotherapy	[67,68,69,70]
Liver cancer and CCA ^1^	HCC organoids: 26%, CCA organoids: 36%	CD8^+^ T cell, TILs, PBMCs	Domes Matrigel	None	[71,72,73]
Breast cancer	87.5%	None	None	None	[74,75]
Prostate cancer	20%	None	None	None	[76]

^1^ HCC, hepatocellular carcinoma; CCA, cholangiocarcinoma.
